# Liquid Biopsy in Lung Cancer: Tracking Resistance to Targeted Therapies

**DOI:** 10.3390/cancers17213474

**Published:** 2025-10-29

**Authors:** Scarlet B. Urtecho, Beatriz Jimenez Munarriz, Mary R. Rabey, Natasha B. Leighl

**Affiliations:** 1 Division of Medical Oncology, Princess Margaret Cancer Centre (PMCC), University Health Network (UHN), Toronto, ON M5G 2M9, Canada; scarletberenice.urtechosuarez@uhn.ca (S.B.U.); beatriz.jimenezmunarriz@uhn.ca (B.J.M.); mary.rabey@uhn.ca (M.R.R.); 2 Temerty Faculty of Medicine, University of Toronto, Toronto, ON M5S 1A8, Canada

**Keywords:** NSCLC, liquid biopsy, ctDNA, target therapies, mechanism of resistance

## Abstract

**Simple Summary:**

Targeted therapies have transformed the treatment of NSCLC with actionable driver mutations, leading to significant improvements in survival and quality of life. Nonetheless, the emergence of acquired resistance is inevitable and remains a challenge for both patients and clinicians. While tissue re-biopsy has long been the cornerstone for uncovering resistance mechanisms, its clinical use is often limited by invasiveness, feasibility, and the inability to fully capture the spatial and temporal heterogeneity of tumors. Liquid biopsy, especially ctDNA analysis, has emerged as a minimally invasive, dynamic tool that allows real-time monitoring of tumor evolution. By enabling the early detection of resistance, by guiding treatment adaptation, and by revealing novel escape pathways, liquid biopsy is reshaping precision oncology. This review highlights the evolving role of liquid biopsy in tracking resistance to targeted therapies in NSCLC and illustrates key examples of the resistance mechanisms exposed through ctDNA.

**Abstract:**

Non-small cell lung cancer (NSCLC) remains the leading cause of cancer-related mortality worldwide. While target therapies have changed the outcomes of patients harboring actionable mutations, resistance inevitably emerges. Circulating tumor DNA (ctDNA) offers a minimally invasive tool for capturing tumor evolution in real time. This approach enables the rapid detection of resistance mechanisms, complements or substitutes for tissue re-biopsy, and reduces the burden of invasive procedures for patients. In this review, we summarize the current evidence on the use of liquid biopsy to uncover resistance mechanisms in patients progressing on targeted therapies, with a focus on its role in dynamic tumor profiling and longitudinal disease monitoring.

## 1. Introduction

Non-small cell lung cancer (NSCLC) accounts for approximately 85% of all lung cancer cases and remains the leading cause of cancer-related mortality worldwide [1]. Over the past two decades, major advances in molecular oncology have led to the identification of actionable genomic alterations (AGAs) and the development of tyrosine kinase inhibitors (TKIs) [2] that have significantly improved outcomes, with overall survival (OS) now measured in years [3]. However, treatment decisions are influenced by improved progression-free survival (PFS), central nervous system (CNS) activity, diverse resistance mutations, drug accessibility, and toxicity-based preferences from both patients and physicians [4].

**Figure 1:**
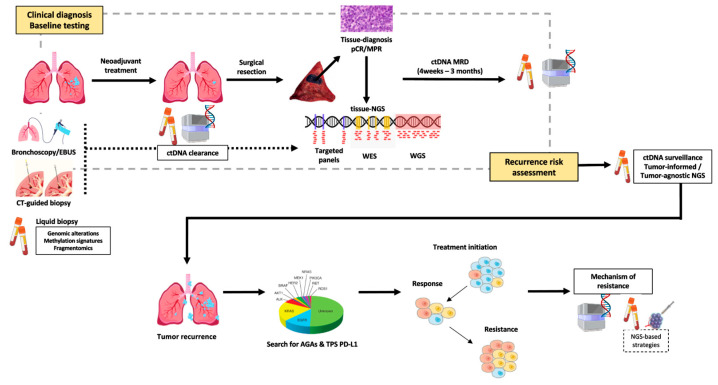
Emerging landscape for ctDNA application in NSCLC.

Resistance to TKI remains a clinical challenge, and tissue biopsy remains the standard for identifying resistance mechanisms after tumor progression. However, repeat biopsies are invasive, time consuming, and may fail to capture tumor heterogeneity [5]. ctDNA analysis has emerged as a minimally invasive tool with broad clinical value that assesses the mechanisms of resistance to targeted therapies, capturing genomic alterations across multiple lesions and subclones, and providing a comprehensive view of tumor evolution (Figure 1). This review explores the expanding role of ctDNA in advanced-stage NSCLC, with a particular focus on its application in monitoring treatment resistance (Table 1).

## 2. Integrating Liquid Biopsy and Tumor Tissue Profiling

Plasma ctDNA analysis and tissue genotyping are complementary tools when assessing the resistance and monitoring efficacy of target treatments [6]. In a large prospective study by Leighl et al. (*n* = 282), ctDNA substantially improved the detection of AGAs in previously untreated metastatic NSCLC, with a 48% increase when compared with tissue sequencing. This benefit was extended to patients with insufficient tissue, baseline unassessed biomarkers, or initially negative mutation results. For FDA-approved targets, concordance between ctDNA and tissue exceeded 98%, with a positive predictive value of 100% and a markedly shorter turnaround time (median 9 vs. 15 days, *p* < 0.0001) [7]. Likewise, Jee et al. showed that paired ctDNA–tissue analysis can confirm tumor relatedness, though concordance is lower in smokers, non-adenocarcinoma histology, and patients with intrapulmonary-only disease. Importantly, ctDNA uncovered additional alterations not detected in matched tissue in up to 25%. These ctDNA-only findings often represented subclonal resistance drivers and carried prognostic weight, being associated with significantly shorter overall survival [8].

Tumor heterogeneity [9]. Rolfo et al. have demonstrated that, when the ctDNA tumor fraction (TF) is ≥1%, additional tissue testing rarely reveals new drivers. In contrast, negative ctDNA results with TF < 1% should prompt reflex tissue testing, particularly in gene amplifications [10]. Different platforms are available and approved for ctDNA analysis; however, evaluation of resistance mechanisms should rely on clinically validated next-generation sequencing (NGS) panels rather than single-gene or polymerase chain reaction (PCR)-based assays [6].

## 3. Defining Tumor Resistance

Definitions of primary (PR) and acquired resistance (AR) are not universally established and vary across targeted therapies and oncogenic drivers. The definition of AR in oncogene-driven NSCLC is often extrapolated from *EGFR*-mutant disease using the Jackman criteria: prior EGFR TKI monotherapy, presence of a sensitizing mutation or clinical benefit, systemic progression on continuous TKI within 30 days, and no intervening systemic therapy [11]. Clinical benefit is typically defined as radiographic response lasting at least six months. While useful in trials, these definitions are challenging to apply in routine practice. On the contrary, PR is generally defined as radiographic disease progression (DP) or lack of clinical benefit within the first six months of TKI therapy.

Current NCCN and ESMO guidelines recommend tissue re-biopsy at progression to identify resistance mechanisms and guide therapy [12,13]. However, Piotrowska et al., in a cohort of 154 patients with *EGFR*-mutant NSCLC, showed that only 39% underwent tissue re-biopsy [14]. These findings suggest that re-biopsies are not often feasible in daily practice and that ctDNA offers a less invasive alternative and better reflects the tumor heterogeneity, though its concordance with tissue NGS may be affected by tumor non-shedding and platform-specific limitations [5].

## 4. Mechanism of Resistance in Targeted Therapies

**Figure 2:**
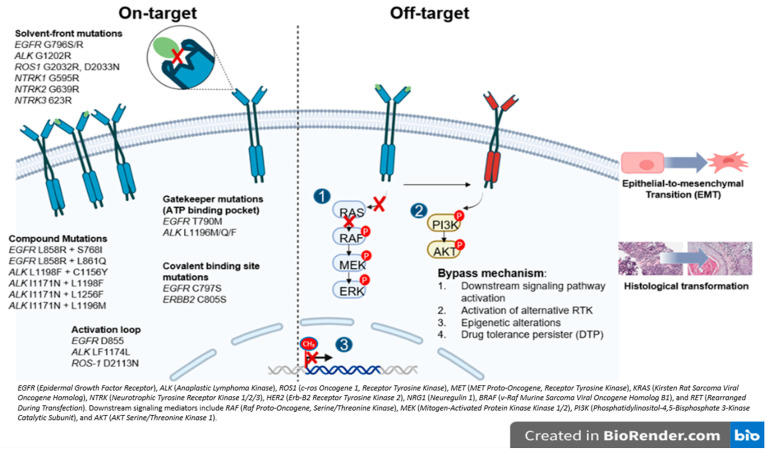
Overview of resistance mechanism associated with oncogenic drivers in NSCLC. The figure illustrates the major actionable alterations and associated signaling pathways. These genes encode receptor tyrosine kinases or downstream effectors that promote tumor proliferation and survival and represent established or emerging therapeutic targets in NSCLC.

Resistance mechanisms are largely categorized as on-target, when the primary target of the drug is altered and off-target, involving activation of alternative or downstream signaling pathways that bypass the original oncogenic driver (Figure 2).

In general, on-target resistance includes secondary mutations in the tyrosine kinase domain (TKD), causing drug resistance by reactivating kinase signaling despite the presence of TKI [15]. The mutations in the TKD can be labeled based on the specific amino acid position affected (Figure 2) [15,16]. The gatekeeper mutations are located in the ATP binding pocket and create steric hindrance, physically blocking access and preventing TKI from effectively binding (i.e., *ALK* L1196M and *EGFR* T790M). In contrast, the solvent-front mutations occur on the outer surface of the ATP binding site exposed to the cytoplasm, where changes in charge or size repel the drug and reduce its affinity (i.e., *ALK* G1202R and *ROS-1* G2032R). The activation loop (A-loop) mutations are found in a flexible region near the active site that regulates the kinase’s transition between active and inactive states (i.e., *ALK* F1174L and *EGFR* D855N). These mutations can lock the kinase in its active form or mimic phosphorylation, resulting in continuous activation and persistent signaling despite TKI treatment [15,17]. Covalent binding site mutations can directly disrupt the attachment of TKIs to their target, preventing irreversible binding and driving resistance (i.e., *EGFR* C797S) [3,18]. Similarly, compound mutations, defined as multiple co-occurring alterations, often emerge after exposure to successive generations of TKIs and become a challenge for targeting. Interestingly, certain combinations may paradoxically re-sensitize tumors to earlier agents, as seen with *ALK* L1198F + C1156Y restoring sensitivity to crizotinib [19]. Beyond the kinase domain, mutations such as *ALK* H694R can also confer resistance by activating parallel or downstream signaling pathways (i.e., STAT3, AKT), producing receptor gain-of-function that differs from classic on-target site resistance [20].

Regarding off-target resistance, the activation of alternative signaling pathways can occur independent of the primary oncogenic driver, and despite effective upstream inhibition, aberrant PI3K/AKT or RAS/MAPK signaling can sustain tumor growth [16]. A similar mechanism underlies the emergence of drug-tolerant persisted (DTP) cells which can survive treatment even as most cancer cells die. In the presence of the drug, these cells adopt a slow-cycling state with altered metabolism and persist through epigenetic adaptations [16,21]. In the same line, epigenetic modifications, particularly DNA methylation, critically regulate gene transcriptional activity, i.e., evidence shows that NSCLC cell lines harboring an unmethylated *EGFR* promoter exhibit increased sensitivity to gefitinib relative to those with promoter hypermethylation, implicating *EGFR* promoter methylation as a potential mechanism underlying off-target AR [22] (Figure 2).

An additional, off-target but non-genomic, mechanism of resistance includes histologic transformation (HT) and the epithelial-to-mesenchymal transition (EMT). HT occurs when the tumors change to a different histological subtype by lineage plasticity. Small cell lung cancer (SCLC) transformation has been reported in 3–14% of patients treated with first- and second-generation EGFR TKI, and in 5–20% of those receiving Osimertinib [23,24] and has also been observed in *ALK-* and *ROS1*-rearranged tumors, suggesting that it is independent of TKI class [25,26]. Transformed SCLC (tSCLC) is characterized by high mitotic activity, expression of neuroendocrine markers, and a high prevalence of *TP53* and/or *RB1* mutations; however, dual *TP53/RB1* loss appears necessary but not sufficient for HT [27,28]. EMT, by contrast, involves epithelial cells acquiring mesenchymal features, enhancing motility and invasiveness. Although the complete mechanism underlying EMT-mediated AR remains unclear, multiple signaling pathways (TGF-*β*, WNT, Hedgehog, Notch, TNF-*α*, and various kinases) have been implicated in repressing *CDH1* (encoding E-cadherin) and promoting mesenchymal marker expression (Figure 2) [29]. Although these genomic alterations suggest a predisposition to HT, tissue biopsy remains the gold standard for diagnosis.

## 5. Assessing Resistance in Targeted Therapies

### 5.1. EGFR

*EGFR-*sensitizing mutations, primarily exon 19 deletions and exon 21 L858R substitutions, occur in ~10–50% of lung adenocarcinomas, with the highest prevalence among non-smoking Asian women [30]. *EGFR* mutations can be detected using PCR-based methods. However, in an RW analysis by Shen et al., *EGFR* PCR showed an 11.3% of FN rate compared with NGS (FoundationOne CDX), in a population with high *EGFR* mutation prevalence [31]. Furthermore, NGS enables the detection of co-mutations such as TP53 or MAPK alterations, which influence response duration and contribute to PR [32].

*EGFR* sensitizing mutations confer marked sensitivity to EGFR TKIs, whether used as monotherapy or in combination with chemotherapy (CT) or EGFR/MET bispecific antibodies (amivantamab), resulting in significant improvements in PFS, ORR and OS, and are recognized as preferred first-line treatment options in both NCCN and ESMO guidelines [12,13]. The FLAURA study first demonstrated the benefit of osimertinib over first-generation TKI, with improved PFS (18.9 vs. 10.2 months) and OS (38.6 vs. 31.8 months) [33].

Consequently, the phase III FLAURA-2 (*n* = 557), assessing first-line osimertinib +/− CT, and MARIPOSA (*n* = 1074), assessing the combination of amivantamab and lazertinib vs. osimertinib monotherapy, further refined first-line strategies for EGFR-mutant NSCLC. In FLAURA-2, osimertinib plus chemotherapy achieved a median PFS of 25.5 months and an OS of 47.5 months. In MARIPOSA, amivantamab plus lazertinib achieved a median PFS of 23.7 months, with median OS not reached at a median follow-up of 37.8 months [34,35].

Primary resistance occurs in roughly 20–30% of patients [15]. To explore the impact of PR, Blakely et al. examined 1122 patients who were profiled using Guardant360 alongside matched tissue sequencing (WES-NGS) and identified at least one additional oncogenic mutation in 92.9% of cases, with *TP53* being the most frequent (54.6%), followed by *PIK3CA* (12.4%) and *BRAF* (11.4%) [32]. Moreover, alterations in *MET*, *NF1*, *CDK4/6*, *CCNE1*, *PIK3CA*, and *APC* were exclusive to patients with PR to osimertinib, and notably, CDK4/6 alterations were associated with markedly reduced mPFS (0.7 vs. 11.2 months; HR 10.3, *p* < 0.005) [32]. Consistent with these findings, Wai et al. reported that the detection of concurrent *EGFR* and *TP53* mutations in pretreatment plasma was associated with poorer prognosis at the start of treatment [36]. The ongoing phase III TOP study aims to assess whether adding CT to osimertinib improves outcomes in patients with concurrent *EGFR* and *TP53* mutations [37].

AR, in terms of first- and second-generation EGFR TKIs, is mostly mediated by the T790M gatekeeper mutation, observed in ~40–60% of cases [23]. Importantly, tumors with T790M often remain sensitive to third-generation TKI, which selectively target both *EGFR* sensitizing mutations and T790M [23]. Multiple studies have proved that ctDNA using PCR or NGS are reliable for detecting T790M, offering comparable outcomes to tissue-based testing. In a study by Oxnard et al., patients with *EGFR* T790M-positive NSCLC (*n* = 58) treated with osimertinib showed similar efficacy whether the mutation was identified in plasma or tumor tissue, with ORR of 63% and 62%, and mPFS of 9.7 months in both groups [38].

Among AR to third-generation EGFR TKIs, the *EGFR* C797S mutation is the most frequently observed on-target alteration [3,18]. In the AURA3 trial (*n* = 419), which compared second line osimertinib vs. platinum-base CT in *EGFR* T790M-positive, C797S emerged in ~10–26% of patients with T790M co-mutations [39]. Notably, the *EGFR* T790M loss was observed in around 50% of patients at progression, suggesting the expansion of pre-existing subclones [40,41]. Interestingly, Arulananda et al. demonstrated, using the oncomine lung cfDNA assay, that when C797S and T790M co-occur on the same allele (in cis), resistance to all EGFR TKIs is observed. In contrast, when they arise on separate alleles (in trans), partial sensitivity to first- or second-generation TKIs may persist, although this phenomenon is uncommon [42].

Consistent with these findings, a pre-specified analysis of the FLAURA study using cfDNA (Guardant360 74-gene and Guardant OMNI 500-gene panels) reported a lower incidence of *EGFR* C797S mutation (~7%), identifying it as the second most common resistance mechanism after *MET* amplification [43]. Rare *EGFR* mutations near the C797 binding site (i.e., C796, L792) or in exon 18 (e.g., L718Q/V, L798I) have also been implicated in resistance to osimertinib [44]. Whereas C796 and L792 substitutions impair drug binding, exon 18 mutations alter ATP binding and may retain sensitivity to earlier-generation TKIs, particularly in the absence of T790M (Figure 1) [45]. In the same context, in the MARIPOSA study, cfDNA profiling using Guardant360^®^ showed that first-line amivantamab plus lazertinib significantly reduced *EGFR* resistance mutations (0.9% vs. 7.9%, *p* = 0.014), lowered complex resistance patterns (27.8% vs. 42.6%), and demonstrated low rates of *TP53/RB1* co-mutations (0.9%) [46].

Regarding the off-target resistance to EGFR TKI, *MET* amplification is the most common bypass mechanism, observed in ~5–22% of cases, with higher prevalence in tumors harboring exon 19 deletions. Reported rates vary according to the criteria used to define amplification (i.e., MET copy number ≥5 or MET/CEP7 ratio ≥2) and the sensitivity of ctDNA assays [47]. In the AURA3 trial, *MET* amplification was identified in 19% of cfDNA samples at progression, often co-occurring with C797S, CDK6, or *BRAF* amplifications, suggesting a possible shared chromosomal origin [39]. More recently, the MARIPOSA study reported a low incidence of *MET* amplification with amivantamab plus lazertinib compared with osimertinib (4.4% vs. 13.6%, *p* = 0.017), while rates of other off-target mechanisms were similar [46].

*HER2* amplification is an uncommon off-target AR mechanism to EGFR TKIs, detected in ~1–12% of cases depending on treatment line and frequently co-occurring with other alterations [48]. *BRAF V600E* mutations have also been reported in ~3% of patients at progression in both AURA3 and FLAURA studies [39]. Other mechanisms include *MAPK1* overexpression and cell-cycle pathway alterations, together present in ~12% of cases [43]. Moreover, data from both the multi-institutional cohort and the prospective ELIOS study highlight the complementary roles of tissue and plasma in profiling resistance to first-line osimertinib. Tissue biopsy identified resistance in ~30% of cases (most often HT or *MET* amplification), while ctDNA detected resistance in ~15%, primarily secondary *EGFR* mutations, with notable discordance between the assays. Together, these findings support ctDNA as a practical, non-invasive triage tool, while reinforcing tissue biopsy as the gold standard for capturing histology and copy number-driven resistance mechanisms [49].

ctDNA has also been explored as a predictive biomarker and a tool to guide subsequent treatment strategies in *EGFR*-mutant NSCLC. In the phase II APPLE trial (*n* = 156), ctDNA-guided sequencing of gefitinib followed by osimertinib achieved comparable PFS to upfront osimertinib, though with more CNS progression, reinforcing the benefit of third-generation TKIs as standard first-line therapy [50]. Exploratory analyses of FLAURA also showed that early ctDNA clearance (at 3–6 weeks) correlated with numerically longer PFS, suggesting its potential as an early predictor of treatment response [51].

#### 5.1.1. EGFR Uncommon Mutations

Uncommon *EGFR* mutations, accounting for 10–20% of cases, include point mutations in exon 18 (G719X), exon 20 (S768I), and exon 21 (L861Q), and can be detected in tissue or plasma using PCR or NGS [41]. Afatinib was the first EGFR TKI to demonstrate efficacy against uncommon *EGFR* mutations, with pooled analyses from LUX-Lung trials showing ORRs of 78% (G719X), 56% (L861Q), and 100% (S768I) [52]. More recently, osimertinib has shown meaningful activity, with the ARTICUNO (*n* = 65) study reporting an ORR of 45% and median PFS of 11 months [53]. Promising results from the interim analysis of the Canadian phase II OCELOT trial demonstrated a confirmed ORR/DCR for evaluable patients with G719X (*n* = 11), L861X (*n* = 3), and S768I (*n* = 5) mutations of 45%/73%, 100%/100%, and 60%/100%, respectively, when treated with osimertinib in the first line (cohort B) [54].

Regarding acquired resistance to uncommon *EGFR* mutations, Pang et al. (*n* = 106) analyzed patients with G719X, L861Q, or S768I using tissue or plasma ARMS-PCR and NGS. The most frequent mechanisms of resistance to afatinib included *EGFR* T790M (11%), *FGFR1* amplification (11%), and *MET* amplification (11%), followed by *CDK4* amplification (7%), *PIK3CA* mutation (7%) [55]. These findings highlight a rational for tumor sequencing in patients progressing to afatinib.

#### 5.1.2. EGFR Exon 20 Insertions

*EGFR* exon 20 insertions (ex20ins) present in ~0.7–6% of EGFR-mutant NSCLC, represent a heterogeneous subgroup with limited sensitivity to EGFR TKIs [56,57]. The detection is possible with PCR or NGS; however, a large retrospective study by Ou et al. showed that PCR would have missed over 40% of ex20in variants identifiable by NGS [58].

The phase III PAPILLON study (*n* = 308) established amivantamab plus chemotherapy as a first-line standard for *EGFR* ex20in NSCLC, showing superior mPFS (11.4 vs. 6.7 months; HR 0.40, *p* < 0.001), ORR (73% vs. 43%), and DOR (9.7 vs. 4.4 months) over CTlone [59]. In addition, the ctDNA analysis (Guardant360) showed higher ex20Ins clearance by cycle 3 day 1 in the amivantamab arm (69% vs. 45%), correlated with improved mPFS (12.2 vs. 6.8 months; HR 0.26). The benefit was also observed in non-clearers (9.8 vs. 4.8 months; HR 0.55), supporting ctDNA clearance as an early response biomarker [60].

In the phase II WU-KONG6 study (*n* = 97), sunvozertinib, a fourth-generation *EGFR* TKI, achieved an ORR of 61% in post-platinum EGFR ex20in NSCLC [61]. ctDNA analysis showed improved outcomes in patients with undetectable ctDNA (ORR 68% vs. 46%; PFS 7.4 vs. 5.5 months), with early molecular clearance correlating with clinical benefit. ARs, including C797S and G724S, were detected at progression [62].

### 5.2. ALK

*ALK* rearrangements occur in approximately 4% of NSCLC cases, typically in younger never- or light smokers, most often with adenocarcinoma and rarely with other oncogenic drivers except *TP53* [63,64]. Immunohistochemistry (IHC) combined with NGS (either tissue or LB) is preferred to maximize detection and enable multi-gene profiling [65,66]. In the Blood First Assay Screening Trial (BFAST; *ALK*+ *n* = 119), blood-based NGS detected *ALK* fusions in 98.6% of cases and achieved an ORR of 87.4%, comparable to the 82.9% ORR observed in the ALEX study, and reported real-world data [67,68]. This study supports the high concordance between tissue and plasma NGS for both common and novel *ALK*-fusions [69].

**Figure 3:**
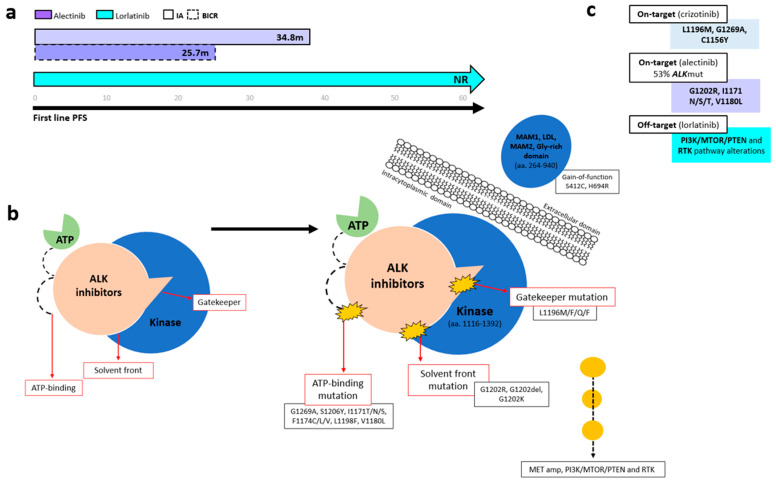
Progression-free survival and resistance mechanisms across *ALK* tyrosine kinase domain regions. (**a**) Kaplan–Meier curves showing PFS among patients with *ALK*-rearranged NSCLC treated with different generations of ALK TKIs. (**b**) Schematic representation of resistance mutations occurring at solvent-front and gatekeeper regions within the ALK kinase domain. (**c**) Summary of the most common *ALK* resistance mechanisms associated with first-, second-, and third-generation ALK TKIs.

Several ALK-TKIs have been approved for first-line therapy of *ALK*-positive NSCLC, including second-generation agents (alectinib, brigatinib, ensartinib and iruplinalkib in China) [68] and the third-generation TKI lorlatinib [70]. In the phase III CROWN trial (*n* = 296), lorlatinib significantly improved PFS over crizotinib, with mPFS NR versus 9.3 months (HR 0.27, 95% CI 0.18–0.39). At 5 years, investigator-assessed PFS remained unreached, with 60% of patients progression-free (Figure 3a) [70,71,72]. Based on the CROWN trial, lorlatinib has become a standard-of-care first-line option in metastatic ALK+ NSCLC; however, a major concern in *ALK*-positive NSCLC is disease progression following first-line lorlatinib. In the 5-year update of the CROWN study, ctDNA analysis using the 74-gene Guardant360 panel from 31 samples from the end-of-treatment (EOT) in the lorlatinib group revealed no emergent *ALK* resistance mutations. Instead, bypass mechanisms (*PI3K/MTOR/PTEN* and *RTK* pathway alterations) (*n* = 29) were the predominant resistance drivers (Figure 3b) [72].

Regarding treatment options, preliminary data from the NVL-655 study with neladalkib, a fourth-generation ALK TKI, showed an ORR of 64% in lorlatinib-pretreated patients with compound *ALK* rearrangement, with all responders maintaining a DOR ≥ 6 months (mDOR NR, 95% CI, 6.9–NE) [73]. Furthermore Mok et al. reported that subsequent systemic therapies after lorlatinib discontinuation provided additional clinical benefit and prolonged PFS2 [74]. Together, these findings support the potential benefit of next-generation ALK TKI and subsequent systemic therapies for extending clinical outcomes even when a percentage of AR to 3rd-generation ALK-TKIs can be off-target.

ctDNA not only enables detection of *ALK* fusions and resistance mutations but also provides prognostic insights during ALK TKI treatment. In a longitudinal study by Kwon et al. (*n* = 92) using Guardant360, the absence of ctDNA at baseline was associated with markedly longer PFS (36.1 vs. 11.4 months, *p* = 0.004) and OS (NR vs. 29.3 months, *p* = 0.02). Furthermore, ctDNA clearance at 2 months correlated with superior outcomes, with median PFS of 25.4 versus 11.6 months (*p* = 0.001) and OS NR versus 26.1 months (*p* = 0.03) [75]. Similarly, an exploratory analysis from the phase III CROWN study (*n* = 296) showed that a reduction in VAF at 4 weeks was associated with response evaluation criteria in solid tumors (RECIST) response in both treatment arms (Table 1) [76]. Moreover, patients with a decrease in mean VAF at week 4 had a confirmed ORR of 75.8% with lorlatinib and 53.3% with crizotinib. In the lorlatinib arm, the HR for dVAF ≤ 0 versus > 0 was 0.50 (95% CI: 0.23–1.12; mPFS NR in either group) [76]. In later line settings of *ALK*-positive NSCLC, Shaw et al. (*n* = 121) showed that early ctDNA dynamics predict lorlatinib efficacy in pretreated patients. Patients with a reduction in VAF (dVAF < 0) showed greater tumor response (26% vs. 12% reduction, *p* = 0.04) and had significantly lower dVAF if they achieved complete response (CR)/partial response (rPR), compared with stable disease (SD) or progressive disease (PD). Importantly, early ctDNA reduction correlated with improved outcomes, with longer median PFS (6.6 vs. 2.6 months; HR 2.6) and OS (18.0 vs. 8.6 months; HR 2.0) [77].

In patients initiating first-line crizotinib therapy, lorlatinib demonstrated efficacy for crizotinib-refractory *ALK*-positive NSCLC in a phase II trial that included cohorts with (*n* = 32, EXP3A) or without (*n* = 27, EXP2) prior chemotherapy [78]. The trial reported an ORR of 69.5%, with a CNS ORR of 87%, and an mPFS NR [78]. These outcomes were consistent across patients, irrespective of the presence of secondary *ALK* mutations, as detected through plasma or tissue genotyping [79]. AR differs with sequential ALK TKIs, with the *ALK* G1202R mutation emerging far more often after second-generation inhibitors (21% with ceritinib, 29% with alectinib, and 43% with brigatinib) than after crizotinib (2%) [80]. In a phase II lorlatinib study (*n* = 198), the most common post-crizotinib mutations detected using plasma (Guardant360) and tissue were G1269A, L1196M, and F1174C, whereas G1202R/del predominated after second-generation TKIs (Figure 3b,c). Lorlatinib showed notable activity against G1202R/del (ORR 57%, median DOR 7.0 months, median PFS 8.2 months) and remained effective across other resistant mutations, with ORRs of 42% for F1174X, 67% for L1196M, and 89% for G1269A [79].

Finally, some *ALK* compound mutations, like L1198F + C1156Y confer resistance to lorlatinib through steric interference with the drug binding but paradoxically enhance binding to crizotinib and resensitize cancer cells to polder ALK inhibitors [19]. In addition, some compound mutations that induce lorlatinib resistance, i.e., I1171N + L1198F, I1171N + L1256F, or I1171N + L1196M, have shown in vitro activity to gilteritinib [81], a multi-kinase inhibitor approved for treating FLT-3-mutant acute myeloid leukemia, an interesting field to explore in vivo.

### 5.3. KRAS

**Figure 4:**
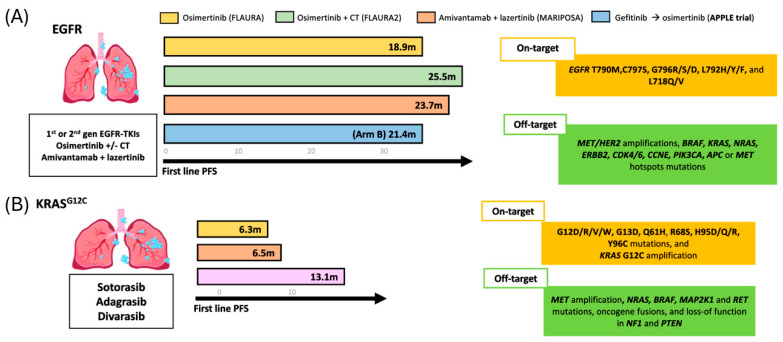
Progression-free survival (PFS) outcomes for and *EGFR* and *KRAS*-mutated NSCLC treated with TKIs. (**A**) rPFS across different first-line treatment in patients with *EGFR*-sensitizing mutations. (**B**) rPFS observed with three distinct second-line therapeutic in KRAS mutated population.

Somatic *KRAS* mutations (*KRAS*m) occur in 30% of NSCLC cases, with G12C representing ~13% [82]. Mutations at codons G12, G13, or Q61 impair KRAS GTPase activity, leading to constitutive activation and persistent downstream signaling via the *MAPK* and *PI3K-AKT* pathways. *KRAS* G12C has a high GTP hydrolysis rate, favoring the inactive GDP-bound state. Inhibitors are most effective in the active GTP-bound form, supporting the efficacy of KRAS OFF inhibitors. FDA-approved *KRAS* G12C inhibitors for pretreated NSCLC include sotorasib (May 2021) and adagrasib (December 2022) [83,84,85]. Figure 4 illustrates the PFS outcomes for *KRAS*- and *EGFR*-mutated NSCLC across first- and second-line treatment.

Molecular response by ctDNA has emerged as a promising biomarker in *KRAS*-mutant NSCLC. In a RW study of sotorasib, Passiglia et al., (*n* = 32) found that ctDNA clearance correlated with better outcomes, including higher ORR (80% vs. 8%, *p* < 0.001), longer mPFS (7.9 vs. 2.8 months, *p* < 0.001), and improved mOS (16.8 vs. 6.4 months, *p* < 0.001) [86]. Likewise, an increase in mVAF anticipated radiological disease progression in 70% of patients who were evaluable at the resistance time point [86]. Similarly, Paweletz et al., reported consistent results in patients receiving adagrasib in the KRYSTAL-1 trial. Using ddPCR, 89.7% of patients (35/39) showed >90% reduction in *KRAS* G12C ctDNA, and 84.6% (33/39) achieved complete ctDNA clearance by cycle 2. Complete ctDNA clearance at cycle 2 was associated with a higher ORR (60.6% vs. 33.3%), while clearance at cycle 4 correlated with longer OS (14.7 vs. 5.4 months) and improved PFS (HR = 0.3) [87] (Table 1). These data support ctDNA as a biomarker of response for *KRAS* inhibitors.

Regarding the AR mechanism found in ctDNA for *KRAS-*mutant NSCLC patients. In a cohort of adagrasib-treated patients (*n* = 27), Awad et al. identified putative resistance mechanisms in 45% (17/27 pts), with 18% (7/17) harboring multiple alterations [88]. On-target resistance included *KRAS* alterations in G12D/R/V/W, G13D, Q61H, R68S, H95D/Q/R, Y96C, and *KRAS* G12C amplification, while off-target mechanisms involved *MET* amplification, mutations in *NRAS*, *BRAF*, *MAP2K1*, and *RET*, oncogenic fusions, and loss-of-function in *NF1* and *PTEN* [88]. Likewise, in the phase I/II CodeBreaK100 study, plasma profiling of sotorasib-treated NSCLC (*n* = 67) showed new acquired alterations in 28% of patients, with *RTK* pathway changes being most common. Despite this, clinical activity remained meaningful, with ORR 41%, median PFS 6.3 months, and median OS 22.5 months [89].

Newer KRAS “OFF” inhibitors demonstrate encouraging activity but similar resistance patterns. In a phase I study of divarasib in KRAS G12C-mutant solid tumors (*n* = 137; NSCLC *n* = 60), the ORR was 53.4% with a median PFS of 13.1 months. The ctDNA analysis (*n* = 70; NSCLC *n* = 25) showed that all patients achieving partial response had *KRAS* G12C VAF <1% by cycle 3 [90]. Paired ctDNA profiling in patients with PFS > 3 months, was performed to assess the mechanism of AR (*n* = 29, NSLC *n* = 8), 55% of the patients (16/29, NSCL *n* = 3) had at least one possible genomic mechanism of resistance identified, such as *KRAS* amplification, *KRAS* non-G12C mutations, *RTK* and *MAPK* mutations, *PI3K* pathway components, and *RB1* copy number loss [90]. In patients with early progression (PFS < 3 months, *n* = 25), longitudinal ctDNA showed emerging non-G12C RAS variants alongside decreasing KRAS G12C levels, suggesting clonal shifts under treatment pressure. Recently, with longer follow-up in KRAS G12C-mutant NSCLC (*n* = 65), divarasib achieved an ORR of 55.6%, a median duration of response of 18 months, and median PFS of 13.8 months [91].

These findings highlight the potential and rationale for combining *KRAS* G12C OFF inhibitors with upstream inhibitors of RTK, such as SHP2. Results from first-in-class *RAS* multi (ON) inhibitors that block multiple *RAS*-isoforms look promising in targeting acquired *KRAS* resistance [92,93,94].

### 5.4. ROS-1 Rearrangement

*ROS-1* rearrangements occur in approximately 1–2% of NSCLC, with over 20 fusion partners identified. The most common is the *CD74-ROS1* fusion, accounting for nearly half of cases, followed by fusions with *SLC34A2* and *GOPC* [95]. *ROS1* alterations are more common in younger, non-smoking Asian women with adenocarcinoma [96]. *ROS1* fusion testing requires two validation tests due to the potential false positives with IHC. However, fluorescence in situ hybridization (FISH) or tissue/plasma NGS cover most of the cases [12].

Clinical guidelines now recommend crizotinib, entrectinib, talatrectinib or repotrectinib as preferred first-line therapies for metastatic *ROS-1* rearrangement NSCLC patients. Crizotinib was the first approved TKI, based on the PROFILE 1001 study (*n* = 53), which reported an ORR of 72%, mPFS of 19.2 months, and mOS of 51.4 months; however, limited CNS penetration was its major limitation [97]. Entrectinib, approval was based on the pooled data from the ALKA-372-001, STARTRK-1, and STARTRK-2 studies, which offered improved intracranial activity with an ORR of 68%, mPFS of 15.7 months, and mOS of 47.8 months [98]. Notably, ctDNA-positive patients had a significantly shorter mDOR compared with ctDNA-negative patients (5.6 vs. 17.3 months, respectively) (Table 1) [99]. Repotrectinib has demonstrated strong efficacy in *ROS1-*positive NSCLC. In the TRIDENT-1 study, TKI-naïve patients achieved an ORR of 79% and an intracranial ORR of 89% (*n* = 71) [100]. Updated data reported a median OS of 74.6 months in TKI-naïve patients (*n* = 121) and 20.5 months in those previously treated with a TKI (*n* = 107) [101]. Lorlatinib has also demonstrated durable responses, with an ORR of 62% and mPFS of 21 months in TKI-naïve patients, and moderate activity in crizotinib-pretreated individuals (ORR 35%, mPFS 8.5 months) [102]. Taletrectinib has also demonstrated strong activity in *ROS1-*positive NSCLC. In the TRUST-I trial (*n* = 173), the cORR was 90.6% in TKI-naïve patients and 51.5% in those pretreated with crizotinib, with meaningful intracranial responses [103]. Updated results showed a cORR of 85.2% in the TKI-naïve cohort, with median DOR and PFS NR at a median follow-up of 20.5 months. In pretreated patients, median DOR was 19.4 months, median PFS 11.8 months, and ORR 61.7% [104]. Zidesamtinib, a highly selective and CNS-penetrant ROS-1 inhibitor, demonstrated, in preliminary data from the ARROS-1 study (*n* = 225), promising activity, with an ORR of 48% and CNS responses in heavily pretreated patients [105]. In the most recent pivotal preliminary data, the TKI-naïve cohort achieved an ORR of 89%, including 9% CR, and an intracranial ORR of 83%. Notably, activity was observed against the *ROS1* G2032R resistance mutation in pretreated patients, with an ORR of 54% and >6-month DOR in 79%. Responses were also reported in other resistance variants, including G1957A, L1982V, S1986F, F2004C/V, G2032K, and D2033N [106].

On-target AR within the ROS1 TKD has been reported in 8–46% of cases. In a study of 55 *ROS1*-positive NSCLC patients (47 post-crizotinib, 32 post-lorlatinib), Lin et al. identified G2032R as the most frequent solvent-front mutation, occurring in 41% and 32% of cases, respectively. Off-target mechanisms were also reported, including *MET* amplification (2.9%), *KRAS* mutations (8.6%, including G12C and Q61H), and *NF1* alterations (7%, all in crizotinib-resistant tumors) [107].

Consistent with tissue-based findings, Dagogo et al., evaluated concordance between plasma ctDNA (Guardant360) and tissue biopsy for *ROS1* fusion detection in NSCLC, both at diagnosis and after progression on crizotinib. At diagnosis, concordance was 100% in seven matched samples. At progression, plasma sensitivity dropped to 50%. Among positive cases, 33% had *ROS1* TKD mutations, with G2032R being the most common. Additional alterations were found in 11% of cases, including *BRAF* V600E and *PIK3CA* E545K mutation [95]. Mezquita et al. analyzed ctDNA in 27 *ROS1*-positive NSCLC patients using the InVisionFirst Lung assay (a targeted amplicon-based platform). Among those who progressed on ROS1 TKI, the G2032R resistance mutation was found in 30%. Interestingly, patients without detectable ctDNA mutations at progression had significantly longer mOS of 105.7 months [108].

Regarding the mechanism of resistance obtained from clinical trials, in the TRIDENT-1 study, early ctDNA analyses revealed no acquired *ROS1* resistance mutations in TKI-naïve patients treated with repotrectinib [100]. Likewise, in the lorlatinib phase I/II study, *ROS1* rearrangements were detected in 15% of pretreated patients using ctDNA, including G2032R, L2026M, and I2025I. Notably, responses to lorlatinib were observed in 27% of patients without detectable *ROS1* mutations, but in none of the patients harboring KD mutations [102]. For Off-target resistance following treatment with crizotinib, entrectinib, and lorlatinib in *ROS1-*positive NSCL, *MET* amplification is the most frequent mechanism. However, *TP53* mutations, *BRCA2* loss, and amplifications in *EGFR* and *FGFR1*, has also been described [107]. In one case report of lorlatinib resistance, plasma ctDNA analysis using Guardant360 revealed an *SLC34A2*-ROS1 fusion alongside *MET* D1246N, *TP53* V173fs, and copy number gains in *EGFR* and *FGFR1*, alterations that were not detected by tissue-based NGS or FISH, highlighting the added sensitivity of liquid biopsy [109].

### 5.5. BRAF

*BRAF* mutations occur in approximately 4% of NSCLC, with *BRAF* V600E being the most prevalent and most common, found in women and non-smokers [96]. Concurrent genetic alterations are common, found in up to 90% of cases. Co-mutations such as *TP53*, *STK11*, *KRAS*, *NF1*, and in various TK receptors have been identified in both tissue and plasma samples. *BRAF* V600E tumors are more frequently associated with *SMAD4* and *PIK3CA* alterations, whereas non-V600E mutations are linked to *KEAP1*, *NF1*, *MET*, *RICTOR*, *KRAS, MYC*, and *STK11* [110]. *BRAF* V600E mutations can be detected in cfDNA using ddPCRor NGS, with high concordance to tumor samples [110].

The approval of dabrafenib plus trametinib (D + T) in 2017 marked the first targeted therapy for NSCLC with *BRAF* V600E mutations, achieving an ORR of 64% and median DOR of 10.6 months [110,111]. In October 2023, the combination of encorafenib and binimetinib (E+B) was approved as the second BRAF and MEK inhibitor, showing an ORR of 75% with median DOR not reached. Both FDA approvals were supported by non-comparative phase II clinical trials. However, RWD from a French multicenter cohort (IFCT-2004 BLaDE study, *n* = 585) using D+T revealed an ORR of 73.8% in patients receiving second lines or subsequent lines, and 82.9% in those treated in the first line, with an mDOR of 10.6 months and 16.3 months, respectively [112]. These results, consistent with registry data, support the meaningful clinical activity of BRAF/MEK inhibition.

Primary and AR to BRAF TKIs remains a major challenge in NSCLC, in part due to the limited lung cancer-specific data, with most current insights extrapolated from studies in melanoma. However, the most common mechanisms involve *MAPK* pathway reactivation, particularly through *ERK* signaling [113]. In terms of biomarkers, Ortiz-Cuaran et al. showed that persistent ctDNA positivity (VAF > 0.01%) at first radiographic evaluation correlated with significantly worse outcomes in BRAF-mutant NSCLC treated with BRAF TKIs, with shorter PFS (5.3 vs. 12.1 months; *p* = 0.023) and OS (9.4 months vs. not reached; *p* = 0.0004) [114].

The most recent data from the LIBRA study, which explored ctDNA dynamics in *BRAF* V600E-mutant NSCLC treated with first-line dabrafenib plus trametinib, used ddPCR (QX200 Droplet Digital PCR) across all samples and NGS (AVENIO ctDNA Expanded Kit) at baseline and progression. Higher baseline *BRAF* V600E VAF was associated with shorter PFS (*p* = 0.013) and OS (*p* = 0.010), while clearance at first evaluation correlated with significantly improved outcomes (all *p* < 0.001). In particular, biological progression detected in ctDNA preceded radiographic or clinical progression by a median of 4.9 weeks (IQR 1.4–9.8). NGS also revealed a heterogeneous resistance landscape, with baseline *EGFR* and *MET* copy number variations predicting poor survival, and acquired alterations at progression including *NRAS*, *KRAS*, *TP53*, and CNVs in *EGFR*, *MET*, and *HER2* [115].

### 5.6. HER2 (ERBB2) Mutations

HER2 (ERBB2), a member of the ERBB receptor tyrosine kinase family, is altered in ~2–4% of NSCLC, most commonly through exon 20 insertions. These alterations are more frequently observed in female, non-smoking patients with adenocarcinoma [96].

Current guidelines recommend tissue-based DNA NGS for detecting *HER2* mutations and amplifications [12,13]. However, results from the DESTINY-Lung01 and DESTINY-Lung02 studies in previously treated HER2-mutant NSCLC showed that ctDNA testing closely matches tissue testing. In DESTINY-Lung01 Cohort 2, plasma and tissue results had a 91% positive percent agreement (PPA) and 100% negative percent agreement (NPA). DESTINY-Lung02 Arm 1 showed similar results, with an 86.0% PPA and 100% NPA. These findings suggest that ctDNA is a reliable alternative when tissue samples are unavailable [116].

The current standard of care for *HER2*-mutant NSCLC remains platinum-based CT with or without immunotherapy (IT). A pooled analysis by Zhang et al. (*n* = 260) suggested improved outcomes with CT-IT compared with IT alone (ORR 37% vs. 26%, DCR 79% vs. 68%, mPFS 7.1 vs. 5.3 months). Beyond CT, trastuzumab deruxtecan (T-DXd) has demonstrated meaningful activity. In DESTINY-Lung01, T-DXd achieved an ORR of 55%, mPFS 8.2 months, and mOS 17.8 months [117], while DESTINY-Lung02 confirmed robust efficacy at both 5.4 mg/kg and 6.4 mg/kg (ORR 49% and 56%) [118]. These results led to FDA and EMA approval of T-DXd for previously treated *HER2*-mutant NSCLC. The ongoing phase III DESTINY-Lung04 trial is comparing T-DXd to first-line platinum–pemetrexed–pembrolizumab [119].

For *HER2*-mutant NSCLC, sevabertinib, is an oral and reversible TKI targeting both HER2 and EGFR. In the phase I/II SOHO-01 trial (*n* = 78), it achieved an ORR of 70.3% in untreated patients and 35.3% in those previously treated, with a median DOR of 8.7 and 9.5 months, respectively [120]. The ongoing phase III SOHO-02 trial is evaluating sevabertinib, as first-line therapy [121]. As with *BRAF*, this remains a rare population, and current understanding of resistance mechanisms is largely extrapolated from breast cancer, underscoring the need for further investigation.

### 5.7. MET

*MET* alterations, particularly exon 14 skipping mutations (*MET*ex14) and gene amplification, represent important oncogenic drivers in NSCLC. *MET*ex14 occurs in ~3–4% of cases, with higher prevalence in females, smokers, and tumors with sarcomatoid histology, though it has also been reported in squamous cell carcinoma, especially among light or never-smokers.

Clinical guidelines recommend tissue-based DNA NGS for *MET*ex14 detection, though sensitivity may be limited by incomplete coverage of exon 14 and flanking intronic regions [12]. Hybrid capture-based NGS offers broader coverage than amplicon-based methods but can still miss non-canonical splice variants. RNA-based assays, including RNA sequencing and RT-PCR, improve accuracy by directly detecting aberrant transcripts and are particularly useful when DNA results are negative or inconclusive [122]. Liquid biopsy platforms such as Guardant360 and FoundationOne Liquid CDx have also shown high concordance with tissue testing, providing a reliable alternative when tissue is limited or unavailable [123].

*MET* amplifications (METamp) occur in approximately 1–5% of NSCLC cases, more frequently among heavy smokers, and may arise as an EGFR TKI resistance mechanism [124]. It has traditionally been assessed using FISH, with scoring based on either gene copy number (GCN) or the MET/CEP7 ratio. Although no universally accepted cut-off exists, high-level *MET*amp (MET/CEP7 ratio ≥ 4–6 or GCN ≥10) has been associated with clinical responses to MET inhibitors in both treatment-naïve and pretreated patients [125]. In contrast, *MET* overexpression shows poor correlation with *METex14* or *MET*amp, though early data suggest it may predict response to MET TKI for *METex14*-positive tumors [126].

Current clinical guidelines suggest that clinicians may offer capmatinib and tepotinib as first-line therapy in METex14-positive NSCLC. In the GEOMETRY mono-1 study (*n* = 364), capmatinib achieved an ORR of 68% in treatment-naïve and 44% in pretreated *METex14*-positive patients. Response was limited in *MET*amp tumors with copy number < 10 (ORR 7–12%), but was higher when the copy number was ≥10 (29% in pretreated, 40% in untreated). In addition, *METex14* was detected in plasma using FoundationOne CDx, with a PPA of 67.2% compared with tissue. Most false negatives occurred in patients with very low tumor DNA levels (VAF < 0.1) [127]. Similarly, tepotinib showed a 46% ORR and 8.5-month mPFS in the VISION study (*n* = 152), but only in the *METex14* population [123]. These results led to global approvals for both agents in the treatment of *METex14*-positive NSCLC. Ensartinib demonstrated promising activity in the phase II EMBRACE trial, where 31 patients with *MET* exon 14-altered NSCLC were enrolled. Among the 30 evaluable patients, median follow-up was 9.2 months, yielding an ORR of 53.3%, an mPFS of 6.0 months, and mDoR of 7.9 months [128].

The mechanisms underlying PR remain incompletely characterized, but co-occurring genomic alterations appear to influence therapeutic response. Subgroup analyses from the VISION (Guardant360) and GEOMETRY mono-1 (FoundationOne CDx) studies reported frequent co-mutations in *MET*ex14 NSCLC, most commonly involving *TP53*, *MDM2*, and *CDKN2A*. Among these, *TP53* mutations were associated with poorer clinical outcomes [127].

In contrast, AR often involves secondary mutations within the *MET* TKD, particularly at D1228 and Y1230, which interfere with TKI binding [129]. Preclinical studies suggest that type II MET TKIs, which bind the inactive kinase conformation (e.g., cabozantinib, glesatinib), may retain activity against certain resistance mutations. In the VISION trial, Guardant360 ctDNA profiling identified *MET* kinase domain mutations in 22.2% of patients with *MET*-amplified NSCLC at progression, most commonly at D1228, Y1230, and D1231. Off-target alterations were also observed, including *TP53/RB1* loss, *EGFR/HER2* amplifications, and *PI3K/RAS* pathway mutations [130]. In a separate phase II study with capmatinib using Guardant360 assay in patients previously treated with crizotinib, 31% of ctDNA-positive patients harbored acquired *MET* mutations, and additional alterations were identified in the *MAPK* (19%) and *ERBB* (13%) pathways [131].

The biomarker analysis of the EMBRACE trial evaluated ctDNA dynamics across *MET-*specific, canonical, and pan-mutation panels using Illumina HiSeq-X10. Baseline plasma was available for 29 patients, with week 4 samples from 20. Frequent co-alterations included *TP53*, *MUC16*, *HGF*, and *NOTCH4*, with *ATRX/TP53* variants associated with shorter PFS (2.9 vs. 7.5 months). While pan-mutation analysis demonstrated high sensitivity (85%) but poor specificity (31%), *MET-*specific monitoring provided a more balanced profile (50% sensitivity, 90% specificity, 83% PPV). By week 4, ctDNA negativity in the MET-specific group correlated with higher ORR and longer PFS, underscoring its potential to identify true non-responders [132].

### 5.8. RET Fusion

*RET* fusions are found in about 1–2% of NSCLC cases, usually in younger, non-smoking patients with adenocarcinoma [133]. Unlike thyroid cancer, where RET mutations predominate, NSCLC is mainly driven by *RET* fusions, most commonly with *KIF5B* (70–90%) and *CCDC6* (10–25%) partners [12].

*RET* fusion detection can be detected by several methods: IHC, ddPCR, FISH, and both DNA- and RNA-based NGS [134]. Unlike ALK IHC, RET IHC has limited sensitivity (55–65%) and inconsistent specificity (40–85%), making it less reliable. Current ESMO and NCCN guidelines recommend NGS (especially those with hybrid DNA/RNA-based platforms) for detection [12,13].

Selective *RET* inhibitors, selpercatinib and pralsetinib, are approved first-line therapies and have shown robust clinical activity. In the updated LIBRETTO-001 trial, selpercatinib achieved an ORR of 61.5% in pretreated patients, with a median DOR of 31.6 months, PFS of 26.2 months, and OS of 47.6 months; in treatment-naïve patients, ORR reached 82.6%, with a median PFS of 22 months, while OS was NR [135]. Similarly, the phase I/II ARROW study reported an ORR of 59.6% and median PFS of 16.4 months in pretreated patients receiving pralsetinib, and an ORR of 75.4% with encouraging durability in the treatment-naïve cohort [136]. These findings support the idea of selpercatinib and pralsetinib as preferred first-line options for *RET-*fusion-positive NSCLC.

PR and AR to selective RET inhibitors can result from both on-target and off-target genetic changes. Studies using tissue and plasma NGS have identified several mechanisms linked to resistance. In a retrospective series of 18 patients treated with selpercatinib or pralsetinib, *RET* G810 mutations were detected in 10%, *MET* amplification in 15% (including two confirmed by ctDNA), and *KRAS* amplification in one case. *MET* and *KRAS* alterations correlated with shorter PFS (6.3–7.2 months), suggesting a role in early resistance [137]. Similarly, a multicenter study (*n* = 95) reported primary resistance in 23% of patients, with poor responders more likely to harbor *KRAS* G12V or *SMARCA4* mutations (4.5% vs. 0%, *p* = 0.2; and 25% vs. 0%, *p* = 0.04, respectively) suggesting these alterations may play a role in intrinsic resistance to RET-targeted therapies [138].

Secondary resistance to selective RET inhibitors is commonly associated with *RET* G810 solvent-front mutations. In patient-derived xenograft models, *RET* G810S emerged following selpercatinib treatment [139]. ctDNA analysis from the ARROW trial showed that RET fusions were generally retained at progression. However, some patients acquired secondary alterations, including *RET* G810 or L730 mutations, *MET* amplification, and *BRAF* V600E, while *RET* V804 gatekeeper mutations were notably absent [140]. In the LIBRETTO-001 trial, G810X mutations were detected in NSCLC whereas *RET* V804 mutations were observed exclusively in patients with medullary thyroid cancer; however, also reported are patients (26%) with acquired *KRAS*- (G12A/R/V, G13D, A59del), *NRAS*- (G13D, Q61R), or *BRAF*-activating mutations or *MET* or *FGFR1* amplifications [141]. In a retrospective analysis of ctDNA and tissue samples from patients treated with selpercatinib or pralsetinib, Lin et al. reported the emergence of acquired *RET* G810 solvent-front mutations as a key resistance mechanism. Additional alterations identified included *RET* L730, V804, S904F, novel *RET* rearrangements, and amplifications of *MET* and *MYC* [137,138]. Similarly, retrospective studies in *RET* fusion-positive NSCLC and *RET-*mutant medullary thyroid cancer using ctDNA and tissue revealed the emergence of *RET* solvent-front mutations following initial responses to selpercatinib [139].

### 5.9. NTRK

*NTRK* fusions involving NTRK1, NTRK2, or NTRK3 are rare but clinically relevant, occurring in <1% of lung adenocarcinomas. They are more often seen in younger patients with little or no smoking history [12]. Common fusion partners for *NTRK* include *ETV6*, *LMNA*, and TPM3 [17]. Although tissue-based remains the diagnostic gold standard, recent advances in plasma NGS offer a promising non-invasive alternative, with high PPV for detecting *NTRK* fusions [142]. First-generation TRK inhibitors like larotrectinib and entrectinib has significantly improved outcomes in patients that are *NTRK* fusion positive. Both drugs received tumor-agnostic FDA approval (larotrectinib in 2018 and entrectinib in 2019) based on early-phase trials showing durable responses [143]. Larotrectinib achieved an ORR of 79%, with an mPFS of 28.3 months and mOS of 44.4 months across pooled data from three studies (NCT02122913, NCT02576431, NCT02637687) [144]. In NSCLC, the ORR was 75% (9/12), including responses in pretreated patients and those with CNS involvement. Similarly, entrectinib was evaluated in the ALKA-372-001, STARTRK-1 (NCT02097810), and STARTRK-2 (NCT02568267) trials, demonstrating an ORR of 57%, mPFS of 11.2 months, and OS of 21 months [145]. Among NSCLC patients, the ORR was 70% (7/10), with intracranial responses in 50% of those with brain metastases, reflecting its CNS activity.

Resistance to first-generation TRK inhibitors remains a major clinical challenge, primarily driven by acquired on-target mutations. Common resistance mutations affect key regions such as the solvent front (i.e., *NTRK1* G595R, *NTRK3* G623R), gatekeeper residues (*TRKA* F589L, *TRKC* F617L), and at the xDFG motif (*NTRK1* G667C/S, *NTRK3* G696A) [17]. Notably, *TRKA* F589 and *TRKC* F617 mutations parallel resistance hotspots seen in other kinases like ALK, ROS1, and EGFR. Off-target mechanisms including *BRAF* V600E, *KRAS* mutations, and *MET* amp have also been reported [143]. To overcome resistance, next-generation TRK inhibitors (selitrectinib, repotrectinib, taletrectinib, and SIM1803-1A) are under study. Selitrectinib has demonstrated efficacy against solvent-front mutations in preclinical studies and early-phase clinical trials [146]. Repotrectinib, designed to target both solvent front and gatekeeper mutations, is currently being evaluated clinically. While most data come from tissue analyses, emerging evidence suggests that ctDNA can serve as a non-invasive method by which to detect resistance mutations. In a study using the MSKCC ACCESS ctDNA platform, resistance mutations were identified in 8 of 27 patients treated with larotrectinib or entrectinib across various tumor types. Among these, 88% (7/8) harbored on-target alterations—primarily *NTRK1 G595R* and *NTRK3 G623R/E/L*—while one patient had an off-target *BRAF V600E* mutation [147].

### 5.10. NRG1

*NRG1* fusions are uncommon drivers most often found in non-smokers with invasive mucinous adenocarcinoma and tend to have low PD-L1 expression and TMB [96]. Detecting *NRG1* fusions can be difficult due to low frequency, and involve a wide range of fusion partners. Current guidelines suggest starting with pErbB3 IHC and confirming with RNA-based NGS12, [13].

Zenocutuzumab, a bispecific antibody that targets HER2 and HER3, showed promising results in the eNRGy study, with an ORR of 29% in NSCLC and 42% in pancreatic cancer, with DOR 11.1 months and mPFS of 6.8 months [148]. These findings led to FDA-accelerated approval in December 2024 for patients with advanced NSCLC or pancreatic cancer with *NRG1* fusions after prior treatment. Emerging data also suggest that activation of the *NRG1*-ErbB3 pathway may contribute to resistance to other targeted therapies, such as lorlatinib in *ALK*-positive NSCLC [149].

## 6. Future Challenges and Conclusions

ctDNA has emerged as a minimally invasive, clinically informative tool for detecting resistance mechanisms and monitoring response in advanced NSCLC. By analyzing ctDNA from multiple metastatic sites, ctDNA captures spatial and clonal heterogeneity often missed by tissue biopsy. Importantly, ctDNA can uncover resistance alterations weeks before radiologic progression, enabling earlier therapeutic intervention. With its extensive molecular coverage and rapid turnaround, ctDNA is poised to become an essential tool in precision oncology. However, successful integration into routine practice will require continued efforts to standardize assays, improve sensitivity, and validate clinical utility across diverse settings.

## Figures and Tables

**Table 1:** Overview of baseline target mutations and acquired resistance mechanisms detected by circulating tumor DNA (ctDNA).

Resistance Profiles by Mutation Location and Liquid Biopsy Detection							
	Type of Resistance	Resistance Mechanisms	Mutation	Resistant to	Prevalence	Trial	Sequencing Platform
EGFR	On target	Gatekeeper mutation	T790M (exon 20)	1° and 2° G TKI	50–60%	Meta-analysis	Multiples as ctDNA
		Covalent binding site mutation	C797S	Osimertinib	1L: 7	FLAURA 1	73-gene assay or a 500-gene assay; ctDNA (baseline tissue samples tested separately) Guardant360
					2L: 10–26%	AURA 3	73-gene assay ct DNA. Guardant Health
		Solvent-front mutations	G796S/R	Osimertinib	1L-2L: 1%	FLAURA 1 and AURA 3	73-gene assay or a 500-gene assay; ctDNA (baseline tissue samples tested separately) Guardant360
			L792X		3.00%	FLAURA 1 and AURA 3	73-gene assay or a 500-gene assay; ctDNA (baseline tissue samples tested separately) Guardant360
			L798I		1–2%	FLAURA 1 and AURA 3	73-gene assay or a 500-gene assay; ctDNA (baseline tissue samples tested separately) Guardant360
	Off target	Parallel bypass activation	Met amplification	1° and 2° G TKI	5%	Meta-analysis	
				Osimertinib	1L: 7–15%	FLAURA 1	73-gene assay or a 500-gene assay; ctDNA (baseline tissue samples tested separately) Guardant360
					2L: 5–50%	AURA 3	73-gene assay ct DNA. Guardant Health
				Amivantamab + lazertinib	1L: 4.4	MARIPOSA 1	74-gene assay Guardant360 CDx
			Her2 amplification	Osimertinib	1L: 1–2%%	FLAURA 1	73-gene assay or a 500-gene assay; ctDNA (baseline tissue samples tested separately) Guardant360
					2L: 5%	AURA 3	73-gene assay ct DNA. Guardant Health
				Amivantamab + lazertinib	1L: 12.5%	MARIPOSA 1	74-gene assay Guardant360 CDx
		Transformation	SCLC	Osimertinib	1L–2L: 15%	MARIPOSA 1 and AURA3	
ALK	On target	Gatekeeper mutation	L1196M/Q	Alectinib	~6–7%	ALEX	73-gene assay ct DNA. Guardant Health
				Crizotinib	7%	PROFILE 1014	No CTDNA, tissue biopsy (NGS, PCR)
		Solvent-front mutations	G1202R	Lorlatinib	None found	CROWN	73-gene assay ct DNA. Guardant Health
				Brigatinib	21–43%	ALTA-1L	38-gene assay ctDx Lung NGS pane
				Alectinib	29%	ALEX	62-gene assay ctDNA FoundationACT
			E1210K	Brigatinib	29%	ALTA-1L	38-gene assay ctDx Lung NGS pane
		Conformational resistance	I1171T	Alectinib	12%%	ALEX	62-gene assay ct DNA FoundationACT
	Off target	Parallel bypass activation	MET amplification	Crizotinib	12–15%	PROFILE 1014	No ctDNA assay
				Alectinib	~15% in tissue, ~7% in ctDNA	ALEX	62-gene assay ctDNA FoundationACT
				Brigatinib	Rare	ALTA-1L	Most tissue
				Lorlatinib	None found	CROWN	73-gene assay ct DNA. Guardant Health
ROS-1	On target	Gatekeeper mutation	L2026M	Crizotinib, Lorlatinib	Rare		
		Solvent-front mutations	G2032R	Crizotinib	41%	PROFILE 1001 (ROS1)	No CTDNA, Tissue biopsy (NGS, PCR)
				Entrectinib	Rare (<5%)	STARTRK-2, ALKA, STARTRK-1	Mostly tissue; limited ctDNA (Guardant360, local NGS)
			D2033N	Crizotinib	Rare (<1%)	PROFILE 1001 (ROS1)	
		Compound mutation	G2032R + L2086F	repotrectinib	Resistance models		
KRAS	On target	Covalent binding site mutation	Y96C	Sotorasib	Identified in vitro/in vivo	CodeBreak 100 and 101	Guardant360), FoundationOne Liquid CDx
				Adagrasib	11% of post-adagrasib	KRYSTAL-1	Guardant360, custom panels
			R68S	Sotorasib	Identified in vitro;	CodeBreak 100 and 101	Guardant360 (Guardant Health), FoundationOne Liquid CDx
				Adagrasib	Same ~11% cohort	KRYSTAL-1	Guardant360, custom panels
			H95D/Q/R	Adagrasib	~11% of adagrasib-treated patients (unique)	KRYSTAL-1	Guardant360, custom panels
RET	On target	Gatekeeper mutation	V804L/M	Vandetanib		Preclinical + case reports	Tissue biopsy
		Solvent-front mutations	G810R/S/C	Selpercatinib	~7–10% at resistance	LIBRETTO-001	Guardant360, Foundation Liquid CDx
	Off target	Parallel bypass activation	MET amplification	Selpercatinib	~2.7%	LIBRETTO-001	Guardant360, Foundation Liquid CDx
MET	On target	Gatekeeper mutation	G1163R	Crizotinib			Tissue only; no ctDNA data
		Covalent binding site mutation	D1228E/G/H/N	Capmatinib and Tepotinib	~20–30% in small post–type I TKI series	Multiple case/series studies	Tissue NGS; ctDNA rare/limited
			Y1230C/D/H/N/S	Capmatinib and Tepotinib	~20–30% in series	Multiple case/series studies	Tissue NGS; ctDNA rare/limited
		Conformational	L1195V/H1094Y	Crizotinib	Preclinical only		
HER2	On target	Covalent binding site mutation	C805S	Poziotinib	~31% in preclinical clones; identified in resistant tumors	Preclinical (Ba/F3)	Tissue
NTRK	On target	Gatekeeper mutation	TRKC F617L, TRKA G667C, TRKC G696A	Larotrectinib		Clinical/structural studies	Tissue only; no ctDNA data
		Solvent front	V573M	Larotrectinib	Identified in resistant clones	Larotrectinib-resistant tumors (preclinical/clinical)	
		Solvent front	TRKA G595R/L, TRKC G623R/E	Larotrectinib/Entrectinib	Multiple clinical cases	Most commonly acquired on-target mutation post-entrectinib/larotrectinib	Tissue only; no ctDNA data

EGFR: Epidermal growth factor receptor; ALK: Anaplastic lymphoma kinase; ROS1: c-ros oncogene 1 receptor tyrosine kinase; KRAS: Kirsten rat sarcoma viral oncogene homolog; RET: Rearranged during transfection; MET: Mesenchymal epithelial transition factor; HER2: Human epidermal growth factor receptor 2; NTRK: Neurotrophic tyrosine receptor kinase; NGS: Next-generation sequencing; PCR: Polymerase chain reaction; 1L: First line; 2L: Second line.

## Data Availability

No new data were created or analyzed in this study. Data sharing is not applicable to this article.
